# Trauma Care in Nigeria: Time for an Integrated Trauma System

**DOI:** 10.7759/cureus.20880

**Published:** 2022-01-02

**Authors:** Isaac C Okereke, Ubaid Zahoor, Omar Ramadan

**Affiliations:** 1 Trauma and Orthopaedics, The Royal London Hospital, London, GBR; 2 Trauma and Orthopaedics, Whipps Cross University Hospital, London, GBR; 3 Trauma and Orthopaedics, University of Dorset Hospitals NHS, Poole, GBR

**Keywords:** trauma burden, pre-hospital care in nigeria, lmic, inclusive trauma system, trauma system in nigeria

## Abstract

Traumatic injuries are a global health problem. Most first world countries have developed comprehensive trauma systems to provide optimal care for injured patients. A trauma system is a coordinated effort in a defined geographic area that attempts to deliver trauma care to all injured patients and is usually integrated with the local public health system.

The majority of low and middle income countries (LMIC) do not have functional trauma systems. This study aims to critique the status of trauma care in Nigeria and make a case for a re-organisation towards an inclusive trauma system.

The methodology was a review of published articles and available grey literature to assess current practices in Nigeria within the various components of a trauma system, viz., injury prevention, pre-hospital care, acute care, and rehabilitation.

The conclusions from this review suggest that integrating existing major trauma centres (MTCs) with smaller local hospitals within a region into an inclusive, interconnected region-based trauma system is the cheapest and most efficient way to improve Nigeria's trauma care and significantly reduce trauma mortality rates.

## Introduction and background

Globally, traumatic injuries affect nearly 5.8 million people annually and are the leading cause of lost years of life, estimated to result in 500 years of lost productivity annually per 100,000 population [1,2]. The public health implications of trauma are more concerning in lower-middle-income countries (LMIC) like Nigeria, where urbanisation and industrialisation [3] without concurrent developments in the health systems have caused a shift in disease epidemiology towards more chronic illnesses and acute traumatic injuries. The unintended consequence of rapid urbanisation in LMIC countries is the disproportionately higher death rates from trauma than their higher-income counterparts [4]. LMICs account for 90% of the global trauma morbidity and mortality rates, with more than 50% of all injuries occurring in Sub-Saharan Africa (SSA). Trauma kills 68 people per 100,000 in SSA, compared to 6.4 people per 100,000 in higher-income European countries [5]. There are several reasons for this disparity in mortality rates between LMICs and Western Europe, the most significant of which is the imbalance in wealth distribution and investments in healthcare. A noticeable feature of LMICs that further worsens outcomes is the skewed distribution of resources: the most advanced medical facilities and personnel are in major cities. The implication of this is that the inhabitants of rural areas will often never reach these superior treatment facilities. 

Trauma care has evolved over the last five decades with lessons learnt from warfare, medical research, technological advancements in imaging and critical care, and the rapid transfer of trauma victims to appropriate centres for definitive management, leading to improved trauma survival [6]. Evidence from western countries such as the US and UK has shown that when trauma care in a region is organised into a system where severely injured patients are managed at specialist centres that can offer definitive care, mortality and morbidity rates significantly improve [7].

Many different trauma systems have been developed in various countries by the slow adaptation of existing hospital systems; the trauma system is structured around the initial pre-hospital management and triage, in-hospital care, and rehabilitation (associated with teaching and research) of trauma victims within a defined geographic area and integrated into a regional public health system [8]. It is essential to recognise that a trauma centre does not constitute a trauma system. A trauma system is defined as "an organised, coordinated effort in a defined geographic area that delivers the full range of care to all injured patients and is integrated with the local public health system" [9]. The American College of Surgeons describes the functional trauma system following a severe injury to an individual as comprising of emergency services (EMS) dispatch and pre-arrival instructions, EMS field triage and transport (ground or air), trauma system hospital, an interhospital transfer (ground or air), trauma centre and team activation, operating room or interventional radiology, intensive care unit (ICU), general care and early rehabilitation, outpatient or inpatient rehabilitation, home and follow-up care, injury epidemiology and prevention [10].

As with most LMIC countries, there is currently no formal trauma system in Nigeria.

Trauma in Nigeria

Nigeria sits on a 923,786 km^2^ landmass on the Gulf of Guinea in West Africa. Cameroon borders it on the east, while Chad, Niger, and Benin border it on the northeast, north, and west, respectively. With just over 200 million people, it is the most populous African country and is the seventh most populous nation globally. A GDP per capita ranging from $1,026 to $3,986 [11]. Nigeria is an LMIC country with approximately 55% of the population in urban centres and 45% in rural areas with poor access roads, inadequate telephone coverage, GSM connectivity, and non-existent resuscitation facilities [12].

In a systematic review of the epidemiology of injuries in Nigeria by L. Thani, road traffic accidents accounted for 68.4% of trauma admissions, with falls a distant second at 5.5% [13]. Also, Osime and colleagues, in a retrospective study of case presentations to an accident and emergency unit in a midwestern state in Nigeria, found that trauma accounted for 98.4% of presentations, and motor vehicle accidents were the mechanism of injury in 54.3% of the cohort. Both studies found the most frequently occurring diagnosis at presentation to be traumatic brain injury with or without other less severe injuries [14]. Rapidly increasing motorisation with an absence of functional trauma systems, ineffective preventive measures such as speed-limit control, and poor road infrastructure are responsible for this trend. 

Penetrating injuries to the chest occur from gunshot wounds (GSW) and non-gunshot related incidents such as stab wounds, impalements in traffic accidents, and assaults. GSW arises mainly from armed conflicts fuelled by political, ethnic, and religious factors in most developing countries and contributes significantly to Nigeria's trauma burden. For example, Amaefule et al. reported a mortality rate of 11.5% for all trauma admissions at the emergency department of a tertiary health centre in northern Nigeria, about three times the mortality rate in England at 4.1% [14,15]. However, a similar study by Onyemaechi in a tertiary centre in Enugu, South-East Nigeria, recorded mortality rates of 4.5% with an average ISS score of 1-50, which is within the worldwide range of 0.5-6% [16]. The proliferation of small arms from tribal and ethno-religious conflicts in the northern parts of Nigeria may account for the higher rates.

## Review

Methodology

**Figure 1:**
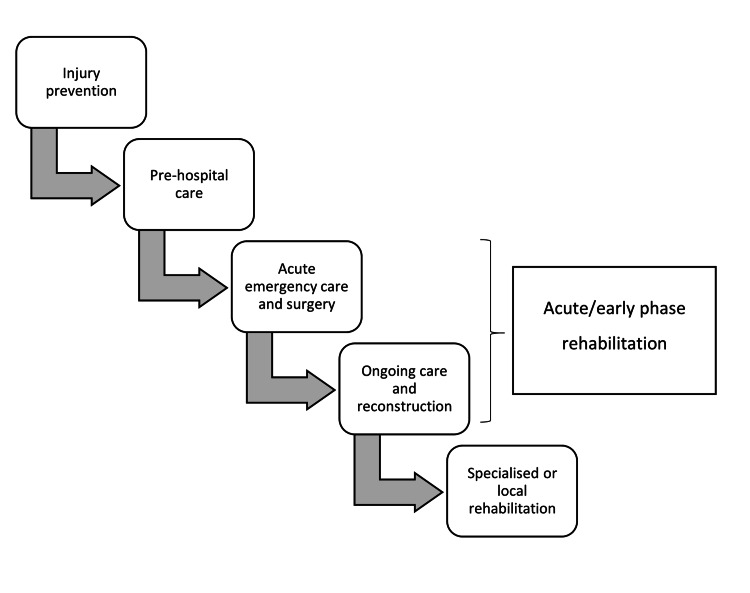
The trauma pathway.

Interrogation of the PubMed, Medline, Cochrane, and EMBASE databases since inception following the Preferred Reporting Items for Systematic Reviews and Meta-analysis (PRISMA) guidelines, using various combinations of the keywords "Trauma," "Nigeria," "Trauma system," and "LMIC." The inclusion criteria were full-text articles in English published in a peer-reviewed journal that reported on the management of trauma in Nigeria, its outcomes, and complications of the Nigerian trauma system. Selected publications had cross-referencing done to obtain relevant articles that met the predetermined criteria of inclusion. Due to insufficient data on trauma systems and a lack of trauma registries in Nigeria, the methodology of this study comprised extracting data from published articles and available grey literature from various LMICs and databases of Google Scholar and international organisations (World Health Organization, United Nations, and World Bank). The resulting data were analysed using the various components of an all-inclusive modern trauma care system, as shown in Figure 1.

Results

Injury prevention is an essential part of a functional trauma system as mortalities are reduced when risk factors for trauma are identified and preventive measures are enacted. For instance, like most other LMICs, Nigeria shares a disproportionately high burden of global road traffic deaths; to address this, the Federal Government of Nigeria created the Federal Road Safety Commission (FRSC) in 1988 to "regulate, enforce, and coordinate all road traffic and safety management activities through public enlightenment, data management, improved vehicle administration, rescue services, and effective patrol operation" [17]. There are also several legislations on speed limits, drunk driving, and the use of seatbelts and helmets that the various state houses of assembly have passed. Nevertheless, the rates of traffic-related deaths in Nigeria are still higher than the global average. This may be attributable to inadequate transport infrastructure, lax enforcement of traffic laws, inefficient vehicle and motorcycle safety regulations, limited road user safety knowledge, and insufficient funding of the FRSC. Further proven strategies for injury prevention include installing speed cameras on motorways and mandating blood alcohol tests for hospitalised trauma patients.

Pre-hospital care of trauma patients is the lifeblood of a functional trauma system. There are two broad categories of pre-hospital care: the "scoop and run" strategy employed in the US, which relies on "paramedics" trained in providing BLS and sometimes ATLSâ to minimise the time spent at the injury scene; and the anaesthetist/intensivist led "stay and play" EMS obtainable in Europe that delivers care en route, including airway management, RSI, chest decompression, IV administration of fluids and drugs [18].

Evidence shows that timely transport and early transfer of injured patients to a trauma centre give more favourable outcomes [19]. The Nigerian health system depends on the direct transfer of patients from the injury scene to the hospital by untrained first responders who are unlikely to give any necessary life support care such as BLS, ATLS®, or basic first aid [20]. There is no national EMS or national pre-hospital health system in Nigeria [21]. Of the 36 states, only Lagos state, with about 9 million people, has an organised ambulance service with one ambulance located in each of its 20 local governments. A study by Venkatraman et al. recorded the average response time of these ambulances as 17 minutes, with a total response rate of 37.1% [22]. The ambulances are effectively a means of transportation, lacking resuscitation equipment and manned by personnel untrained in basic resuscitation protocols or airway management skills [23].

Furthermore, a lack of integration of the ambulance service with the trauma centres leads to suboptimal care of trauma patients and pre-hospital mortality rates that are well above 70% and preventable mortality rates as high as 40% [23,24]. A retrospective study of trauma patients at the Lagos University Teaching Hospital by Ibrahim et al. reported less than a quarter of trauma admissions presenting to the emergency department within the first hour of injury. Only 2.3% of these patients had any form of pre-hospital care [25]. The picture is gloomier for rural dwellers, who often do not make it to the hospital. CNS damage, bleeding, and compromised airways leading to airway obstruction are usually the leading causes of preventable post-trauma mortality in these patients [20].

Nigeria runs a three-tier healthcare system of primary, secondary, and tertiary care. The Federal Ministry of Health (FMoH) develops policies, plans, and programmes that dictate the direction of the National Health Care Delivery System [26]. The primary health care system is coordinated by the local government authorities (LGA) with supervision from the state ministries, which also run secondary health facilities. According to a 2015 BMI report, there were an estimated 3,534 hospitals in 2014. Fifty-four of these were federal tertiary hospitals, comprising 20 teaching hospitals, 22 federal medical centres, 3 national orthopaedic hospitals, the National Eye Centre, the National ENT Centre, and 7 psychiatric hospitals, all under the direct supervision of the FMoH. The healthcare expenditure per capita was $73.9 in Nigeria in 2017. The UK's stood at $3858.7 in the same year [27]. While data on conventional public health issues such as nutrition, HIV/AIDS, malaria, etc., are readily available in Nigeria, there is a shortage of data on trauma and injury management, making it very difficult for trauma advocacy [28]. This may explain the lack of funding by local governments and international donors for Nigeria's trauma epidemic. Acute care and management of trauma patients in hospitals are usually limited by inadequate training and human resources. In Enugu, Nigeria, a study showed poor knowledge of ATLS® protocols among non-specialist doctors involved in trauma care [29].

A functional inter-hospital transfer of injured patients is an essential component of a sound trauma system. However, there is no documented data on protocols for inter-hospital transfer of trauma patients in Nigeria, nor is there a standardised composition of trauma teams or registries. Data on critical care capacity in Nigeria and other low-income countries are marginal due mainly to a shortage of trained critical care professionals, inadequate funding, and poor infrastructure to perform research [30,31].

Bridging the disparities in the outcomes of trauma between affluent and low-income countries like Nigeria is the thrust of the WHO guidelines on trauma care, which identify specific areas of low-income countries' systems that should be targeted for improved outcomes [32].

Rehabilitation after trauma is an indispensable part of trauma care but does not constitute part of the care provided to trauma patients in Nigeria. Trauma hospitals often lack trained physical and occupational therapists and are not adequately equipped with the necessary orthotics and rehabilitation riggings. 

Discussion

With the knowledge that the human and environmental interactions that lead to trauma are essentially like those of many chronic diseases, albeit with differences in timing, trauma is now appreciated as a public health concern. As a result, trauma prevention, like any other public health issue, requires extensive epidemiologic and social planning, as well as meticulous and expertly designed social intervention programs [33,34]. In addition, optimal trauma care requires coordination between local and state systems; therefore, a public health approach is necessary. This is because public health practice methods provide a proven, systematic approach to problem identification and problem-solving by affording a conceptual framework for developing and managing health problems such as trauma systems, with ongoing performance audits and improvement.

Even though the civilian population bears most of the trauma burden, the history of trauma care is inextricably linked to lessons learnt from theatres of war. During the Revolutionary War in the United States, the surgical care of trauma victims was based on principles gleaned from European surgeons like the Hunter brothers and was primarily focused on the treatment of soft tissue injuries and limb amputations [35]. Through the first and second world wars, surgeons understood the use of blood in the resuscitation of trauma victims. During the Korean Conflict and the Vietnam War, the pathophysiology of haemorrhagic shock was better understood, leading to better outcomes and fewer incidences of renal failure in subsequent wars. The 1966 landmark publication by the National Academy of Sciences, "Accidental Death and Disability: The Neglected Disease of Modern Society" [36], outlined a framework to correct deficiencies identified in traumatic injury management and is considered by most scholars as the foundation for the first civilian trauma centres established in the United States in 1966 [37]. By the year 2000, a trauma system had been implemented in every US state. However, differences exist between the European and US trauma systems, which have independently evolved over several decades. Whilst most European countries favour a physician-led pre-hospital care team, in the United States, non-physicians ("paramedics") constitute the emergency response teams. Also, with regards to the in-hospital care of trauma patients, emergency physicians and trauma surgeons provide the initial care for severely injured patients in the US, with the surgeon typically leading the team and assuming responsibility for the patient, as opposed to the anaesthetist/intensivist led trauma team that is favoured by their European counterparts [38]. 

Based on their structural organisation, trauma systems can be classified into "inclusive" and "exclusive" types. In an exclusive trauma system, one hospital acts as a stand-alone major trauma centre (MTC) that houses most surgical specialties and provides specialist care to severely injured patients. There is usually no connection with surrounding hospitals or pre-hospital services, and they act as a funnel rather than a network. Although most US states are now reorganising their trauma systems, it is the predominant trauma system in the US. The framework of trauma care presently available in Nigeria resembles an exclusive system with established government-funded tertiary hospitals serving different populations and providing specialised care. The advantage of this system, as shown in several studies, is the reduction in relative risk of death by over 24%, especially for the most severely injured, when treatment is in an MTC [39-41]. Conversely, the main disadvantage of this system is that it leads to the de-skilling of staff in non-MTC centres and an increased risk of volume overload in the MTCs. Also, in areas with limited resources, such as Nigeria, transport times to the trauma centre may be too long and negatively impact patient survival.

In an inclusive trauma system, all healthcare facilities within a region are involved in the care of injured patients in accordance with their capabilities and available resources. It consists of the implementation of triage and bypass protocols by all hospitals within a network to deliver trauma patients to the most appropriate facility.

Although MTCs play a central role in this network, there is a collaborative approach between all partners in a network. This system relies on a two-way inter-hospital transfer agreement that allows the EMS teams to transfer severe trauma patients to the regional MTC, bypassing local hospitals, and repatriate these patients to their "local" hospital after acute care. The objective is to refer severely injured trauma patients to the MTCs through established triage protocols and allow patients with mild to moderate injuries to be optimally managed in local hospitals. The advantages of this system are that it optimises the resources of the hospitals within a network and matches the level of care required by the patient to a receiving centre's available resources and capacity. In addition, MTCs avoid oversaturation by patients with minor injuries that could otherwise be managed at lower centres. Several studies have shown that inclusive trauma systems have a generally better prognosis for trauma patients and are more beneficial to both patients and health economies than exclusive systems [42].

Trauma systems have been shown to improve the overall survival of severely injured patients. Studies have shown that although exclusive trauma systems improve trauma indices, they are more expensive and challenging to implement, especially in resource-poor countries [42]. Conversely, integrating existing tertiary trauma centres with other medical facilities, thereby forming an inclusive, interconnected region-based trauma system, seems the cheapest and most efficient way to improve Nigeria's trauma care. The establishment of strategies for the organisation of a national trauma care system in Nigeria will have to be formulated by health policymakers with input from medical professionals who will provide research-based guidelines and standards on treating trauma victims [19]. Achieving this requires an initial comprehensive needs assessment of existing medical facilities within a region and specifications of the level of trauma care that can be accessed at each of these facilities. In addition, policymakers will need to evolve triage models and referral systems according to the capacities of each of the hospitals in a region and develop guidelines on nursing care and specific treatment protocols for particular injuries at the different levels of care [43]. This should be followed by education and infrastructure improvements, including establishing an integrated state or region-based EMS dispatch service and formalisation of EMS and emergency physician training. Optimal pre-hospital care is essential considering Nigeria's topography and landscape, where 45% of the population lives in rural areas with poor access roads to urban centres. A functional but straightforward EMS service with well-trained first responders and standardised pre-hospital care, as demonstrated in the 2005 WHO report on pre-hospital trauma care, will considerably reduce death rates [24].

Also, establishing a trauma database with variables adapted to the local environment in Nigeria should be implemented at regional levels nationwide. An example of this is the Kampala Trauma Score (KTS), a validated trauma Injury Severity Score (ISS) adapted for LMIC countries. It differs from the ISS and Revised Trauma Score (RTS), which require more complex calculations and have age specifications that are more in sync with higher-income countries [44]. Efficient compilation of injury data that are simple and standardised across the trauma system is vital for decision-making, health policy formulation, and budgetary planning for a trauma system [45].

Furthermore, routine training for trauma teams should be implemented across states and regions. As documented in several studies, the positive impact of training on the trauma systems in low- and middle-income countries is significant. For example, Gregory et al. reported sustained changes in physical and human resources' availability after implementing a two-year training programme on the WHO Guidelines for Essential Trauma Care for trauma teams [31,46].

Finally, a functional rehabilitation program for trauma patients to optimise people living with disabilities post-injury is an essential component of a trauma system. The WHO estimates that about 15% of the global population lives with a disability, and 80% of them reside in LMIC countries [47]. Barriers to accessing rehabilitation, such as affordability, equipment, and medications [48], have to be addressed when developing a trauma system for Nigeria.

## Conclusions

In this study, the shortfalls in the various components of the trauma system in Nigeria were reviewed. Whilst there has been some progress in the areas of injury prevention through public information and traffic regulation enforcement by agencies such as the FRSC, pre-hospital services and acute trauma care require a complete overhaul and huge capital and human investments to significantly lower mortality rates from trauma. Also, integrating existing tertiary trauma centres with other medical facilities, thereby forming an inclusive, interconnected region-based trauma system, seems the cheapest and most efficient way to improve Nigeria's trauma care.
